# Supplementary material for: Different Synchrony in Rhythmic Movement Caused by Morphological Difference between Five- and Six-armed Brittle Stars
Source: Sci Rep. 2019 Jun 5;9:8298. doi: 10.1038/s41598-019-44808-w (PMC6549144; doi:10.1038/s41598-019-44808-w)
Supplement: Supplementary file 1 — Supplementary Information [file 41598_2019_44808_MOESM1_ESM.pdf]

Different Synchrony in Rhythmic Movement Caused by Morphological Difference between Five- and Six-armed Brittle Stars

Daiki Wakita[1], Yumino Hayase[2], Hitoshi Aonuma[1,3]*

Supplementary Video S1.

Rhythmic movement, “pumping”, in a five-armed individual of the green brittle star *Ophiarachna incrassata* in the aboral side.

Supplementary Video S2.

Rhythmic movement, “pumping”, in a six-armed individual of the green brittle star *Ophiarachna incrassata* in the aboral side.
